# Implementing endoscopy video recording in routine clinical practice: Strategies from three tertiary care centers

**DOI:** 10.1055/a-2592-3338

**Published:** 2025-06-17

**Authors:** Jonas L. Steinhäuser, Tyler M. Berzin, Mark E. Geissler, Cornelius Weber, Nora Herzog, Maxime Le Floch, Stefan Brückner, Jochen Hampe, Sami Elamin, Joel Troya, Alexander Hann, Franz Brinkmann

**Affiliations:** 1 Else Kröner Fresenius Center for Digital Health, Technical University Dresden, Dresden, Germany; 2 39063 Department of Medicine I and Faculty of Medicine, University Hospital Carl Gustav Carus, Dresden, Germany; 3 Center for Advanced Endoscopy, Beth Israel Deaconess Medical Center and Harvard Medical School, Boston, United States; 4 Interventional and Experimental Endoscopy (InExEn), Department of Internal Medicine II, University Hospital Würzburg, Würzburg, Germany

**Keywords:** Quality and logistical aspects, Image and data processing, documentatiton, Training

## Abstract

**Background and study aims:**

Endoscopy video recordings are valuable data for training and deploying artificial intelligence (AI) models. However, collecting these data is challenging and time-consuming, demanding new workflows and robust data management strategies.

**Methods:**

Here, we outline the challenges associated with routinely recording endoscopy data in clinical practice and share experiences and solutions from three endoscopy centers in Germany and the United States.

**Results:**

Each center uses a recording setup tailored to specific needs of that endoscopy unit. Common challenges include integrating with the hospital’s electronic health records, automating video recording, and addressing data privacy concerns. In all cases, having dedicated research staff to manage daily operations has proven essential for successful implementation.

**Conclusions:**

By describing successful strategies, we aim to inspire gastroenterology divisions worldwide to adapt routine video recording for endoscopy procedures, thereby increasing the volume and diversity of datasets necessary for developing clinically impactful AI applications.

## Introduction

Real-world clinical data that are routinely generated in hospitals represent a rich source of information for scientific research and innovation. Development and propagation of “clinical data warehouses” centralizes data organization and streamlines the process of reusing this data for scientific purposes or improving clinical care 1 . Although gastrointestinal endoscopy generates vast amounts of high-definition video data, only a few selected still images are typically archived alongside the written examination report in routine clinical practice. Nevertheless, complete recordings of endoscopic examinations are a valuable resource for teaching and research. For scientific purposes in general and for developing artificial intelligence (AI) models in particular, access to large amounts of real-world endoscopy data is necessary 2 . This endeavor requires implementation of appropriate infrastructure and workflows to integrate endoscopy video recording into routine clinical operations. Here, we share experiences from three large, tertiary care endoscopy centers in establishing endoscopic video recording of routine clinical examinations for scientific purposes. Our aim is to encourage others to join our effort in harnessing the valuable endoscopic videos generated by clinicians to advance the field by leveraging this rich and diverse data.

## Methods

Recording endoscopic videos necessitates capturing the video signal between the endoscopic video processor and the monitor, without compromising the primary clinical feed. Therefore, consultation with the on-site medical technician is necessary to jointly develop a solution for determining at which point in the video signal chain it is most reasonable to capture the endoscopic video for archiving. Depending on the system, manufacturer consultation and adjustments may be required to access the raw video signal before processing. Auxiliary, medical-grade hardware, certified for clinical use, is typically required to process and record the video signal. The recorded video can be output to hard disk (HDD) or network drives and cloud storage.

When recording videos for scientific analysis, ensuring data protection and privacy compliance is crucial, which involves removing identifying information and assigning pseudonyms to the recordings. In Europe, the General Data Protection Regulation (GDPR), and in the United States, the Health Insurance Portability and Accountability Act (HIPAA), provide legal basis for recording and reusing patient data for scientific purposes. In general, recording fully anonymized data involves fewer legal and ethical requirements than handling pseudonymized videos. Some hardware can anonymize videos, excluding patient information (entered manually or obtained from the hospital worklist) from recordings and output filenames, but this can complicate retrospective merging of the endoscopic procedure with the written report and/or other clinical data of the patient. In addition, caution is needed when third parties, such as commercial recording solution providers, handle data, for example when using cloud storage. Also, manual review of the videos is needed to remove any personal data (images of the endoscopy suite, staff, or the patient) from the video before it can be used for scientific purposes. During this essential step, the process of pseudonymization (e.g., by assigning a study ID and renaming files accordingly) can be integrated. This addresses privacy concerns in light of ongoing data ownership debates 3 . Ideally, deidentification and pseudonymization would be managed at the institutional level, but because establishing such processes can take years, it is currently more practical to keep them within the research group. For raw recorded videos, it is advisable to set up a pipeline for semi-automatic preprocessing (i.e., preparing the recordings for future analysis) and management of procedure-related data in a dedicated research database.

Minimizing additional work steps for the endoscopy staff on-site is essential for ensuring compliance with the video recording protocol. Ideally, any additional effort required to facilitate the recording should not interfere with routine workflows in the endoscopy suite. Change management in healthcare and modifying routine workflows within a well-established medical team can be challenging 4 and requires careful preparation and testing before starting data collection. Prior to implementation, it is advisable to explain the proposal and actively seek input from the endoscopy staff. For the recording process itself, clear and concise practical instructions must be provided both verbally and in writing, supplemented by brief instructions (e.g., pictograms) kept close to the recording device. Especially during the initial implementation phase, it is recommended to have a dedicated contact person available that the staff on-site can address in case of uncertainties or problems. It is advisable to routinely check recordings for patterns of failures or non-compliance. Often, errors occur when staff are unsure how to use the recording device and avoid it for fear of mistakes. Identifying this can lead to providing additional instructions as needed.

In addition, the perspective of the examiner should be considered. Endoscopists may be reluctant to have their procedures recorded 5 . It is crucial to communicate transparently the purposes for which the videos are used, ensuring that quality control or evaluation of examiners is not conducted without their informed consent.

## Results

### Dresden

Each examination room in the gastrointestinal endoscopy unit at the University Hospital Dresden, Germany, is equipped with an Ibox Touch (meso international GmbH, Mittweida, Germany) recording device, which connects to the hospital local area network via ethernet. The device receives input from the endoscopic video processor (Olympus Deutschland GmbH, Hamburg, Germany) via a HD-SDI output. For each examination, endoscopy staff manually enters the patient ID and initiates and terminates the recording. The resulting video is automatically exported in chunks of approximately 10 minutes and saved at a resolution of 1920×1080 pixels to a hospital network drive. Following basic quality control, a research assistant assigns a study ID and creates a new entry in a dedicated REDCap 6 database.

Subsequently, a series of Python scripts execute general preprocessing. Semi-automated preprocessing steps include: 1) concatenating video chunks of the recording; 2) cropping the video to retain only the endoscopic video signal by removing extraneous black borders around the video frame; 3) automatically detecting and blurring nuisance text (e.g., text displayed by the recording device or the video processor); and 4) trimming videos to only contain the intraluminal part of the procedure. Only the final step requires human assistance to provide time stamps, or more precisely, frame indices of the relevant video sections to the script.

Recorded ERCP videos are currently being processed and prepared for publication as a publicly available dataset. Analysis of the processed videos shows that their duration ranges from 2 to 113 minutes, with an average length of 30.6 minutes. The videos have resolutions of either 1080 × 1080 or 1232 × 1048 pixels, depending on the endoscope used. According to the corresponding written reports, 32% of the videos capture examinations of patients undergoing their first ERCP, whereas 61% are repeat ERCPs, and in 7% of cases, this information is unknown. A papillotomy was performed in 59% of all cases, and severe bleeding—defined as bleeding requiring additional intervention such as coagulation or clipping—occurred in 8% of videos.

**Fig. 1:**
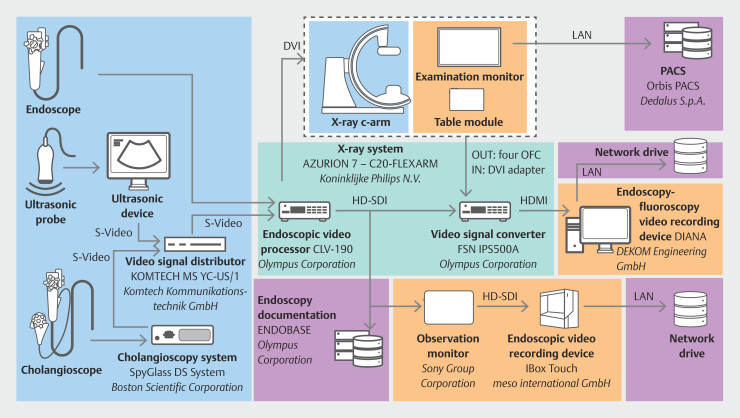
Schematic overview of the endoscopy video recording setup at Dresden. Illustration of signal flow within the endoscopy room, highlighting points of video signal capture. Devices for video signal generation are marked in blue, video processing devices in turquoise, elements responsible for data recording in orange, and data recording destinations in purple.

Also, we implemented a workflow to simultaneously capture the endoscopy and fluoroscopy signal side-by-side e.g., during endoscopic retrograde cholangiopancreatography (ERCP). In contrast to our current approach, this task requires a video recording device capable of handling two independent video sources simultaneously, for which we use the DIANA (DEKOM Engineering GmbH, Hamburg, Germany) video recording system ( Fig. 1 ).

### Boston

**Fig. 2:**
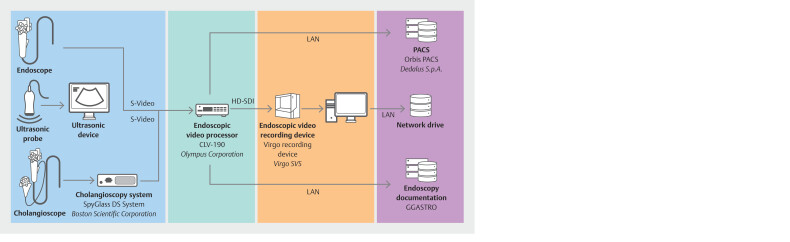
Schematic overview of the endoscopy video recording setup at BIDMC. Illustration of signal flow within the endoscopy room, highlighting points of video signal capture. Devices for video signal generation are marked in blue, video processing devices in turquoise, elements responsible for data recording in orange, and data recording destinations in purple.

The endoscopy unit of the Beth Israel Deaconess Medical Center (BIDMC), a Harvard Medical School teaching hospital, employs multiple recording technologies across 18 endoscopy rooms, enabling a comparison of various systems. All rooms are equipped with Olympus endoscopic video processors (Olympus America Inc., Center Valley, Pennsylvania, United States) and Virgo recording devices (Virgo Surgical Video Solutions Inc., San Francisco, California, United States), which capture 1080p videos and store them on a commercial cloud server ( Fig. 2 ). The Virgo system utilizes AI to automate initiation and termination of recordings based on scope insertion and removal, although manual trimming by research staff is often necessary due to limitations of the AI algorithm. The Virgo system lacks integration with the hospital electronic health record (EHR) system, necessitating manual mapping of recorded videos to patient identities based on procedure room and start time. This task can be performed retrospectively by research staff independently of clinical personnel. In addition, research staff can also match the videos to downloaded procedure reports, histology results, and preoperative, intraoperative, and postoperative reports obtained from the EHR. Videos and their corresponding endoscopy reports are subsequently stored in a Microsoft Teams cloud, with patient information documented in a secure Excel spreadsheet. Although the Virgo system demonstrates proficiency in single-stream recording, capture of multiple data streams (e.g., concurrent fluoroscopy and endoscopy sources), has presented challenges.

In addition to the main recording system, two other systems are employed in the BIDMC endoscopy unit. An Olympus Image Stream (Olympus America Inc., Center Valley, Pennsylvania, United States) system is used in the advanced endoscopy unit because of its capability for handling multiple datastreams simultaneously. However, this older system requires endoscopy staff to enter patient data by hand and to manually initiate and terminate the recording process. This approach was found to be less reliable, because staff may be too busy to enter patient data and do not reliably initiate and terminate the recording, resulting in loss of recordings or requiring manual trimming of video recordings. A third video recording system, the AIDA system (Karl Storz SE & Co. KG, Tuttlingen, Germany), is integrated with the hospital EHR and, therefore, able to obtain patient information and intraprocedural matching of the recording to patient records. However, it is not an “always-on” recording either, necessitating staff to manually initiate and terminate each recording.

A first subset of endoscopy video recordings has been processed and collected in a dataset for ERCP 7 .

### Würzburg

The endoscopy unit at the University Hospital Würzburg, Germany, and the eight associated academic and non-academic centers participating in clinical AI evaluations, exhibit a diverse range of setups, workflows, and endoscopic processors handling both digital and analog signals. Beyond mere recording, our objective encompassed real-time application of one or more in-house developed AIs on the endoscopic video feed, presenting the AI output to the examiner on the primary or secondary display with minimal latency.

**Fig. 3:**
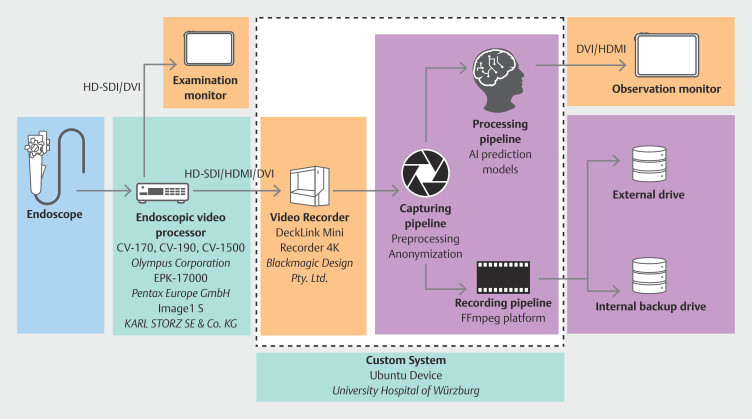
Schematic overview of the endoscopy video recording setup at Würzburg. Diagram illustrating signal flow within the endoscopy room, highlighting the points of video signal capture. Devices for video signal generation are marked in blue, video processing devices in turquoise, elements responsible for data recording in orange, and data recording destinations in purple.

Our system comprises a computer running Ubuntu (Canonical Ltd., London, UK), equipped with a DeckLink Mini Recorder 4K (Blackmagic Design Pty. Ltd., Port Melbourne, Australia) video capture card and a MSI-GeForce RTX3080Ti (NVIDIA Corporation, Santa Clara, California, United States) graphics card ( Fig. 3 ). Utilizing this capture card facilitates acquisition of SDI and HDMI signals without the necessity for conversion, thereby eliminating potential latency. Our custom-developed C++ application for image stream acquisition enables parallel execution of distinct threads: image capture, processing, and display. This architecture ensures imperceptible image delay. Within the capturing pipeline, we have the option to directly anonymize data through image cropping, overlaying a black bar on the patient information, or selective blurring. The video file is securely stored on an encrypted external HDD and redundantly on an internal HDD for backup purposes.

To ensure recording commences only after informed consent has been obtained, we developed an associated device featuring a button LED that indicates active recording. The system with all hardware components is described in detail in Lux et al. 8 . The software framework is freely available for research purposes from https://ukw.de/inexen .

Table 1 provides an overview of the different recording strategies employed at the three institutions.

**Table:** **Table 1** Comparison of recording setups among three centers.

	Dresden	Boston	Würzburg
Endoscopy recording device(s)	Ibox Touch	Virgo, AIDA, Image Stream	Custom grabber card-based recording
Fluoroscopy recording device(s)	DIANA	Image Stream, Virgo	Not applied
Procedures	ERCP, ESD, POEM	EGD, Colonoscopy, ERCP, EUS, ESD, POEM	EGD, Colonoscopy, EUS
Recording destination	Hospital network drive	Cloud, hospital network drive	Hospital network drive
Research database	REDCap	Microsoft Teams	Custom SQL database
Dedicated staff	Research assistant	Research assistant	Research assistant
EGD, esophagogastroduodenoscopy; ERCP, endoscopic retrograde cholangiopancreatography; ESD, endoscopic submucosal dissection; EUS, endoscopic ultrasound; POEM, peroral endoscopic myotomy. Ibox Touch (meso international GmbH, Mittweida, Germany). DIANA (DEKOM Engineering GmbH, Hamburg, Germany). Virgo (Virgo Surgical Video Solutions Inc., San Francisco, California, United States). AIDA (Karl Storz SE & Co. KG, Tuttlingen, Germany). Image Stream (Olympus America Inc., Center Valley, Pennsylvania, United States).			

## Discussion

### Dresden

Although we have successfully recorded over 1300 ERCPs and other procedures over the past 3 years using our method, several opportunities for improvement exist. Manual entry of patient IDs introduces additional workload for staff and is error-prone; integrating the recording device with the hospital DICOM server could streamline this process. Furthermore, manual editing of videos to retain only intraluminal sections is labor-intensive, particularly during instances of frequent endoscope retraction (e.g., during stent replacement); training a convolutional neural network could automate removal of extraneous footage. Finally, because the video signal source (i.e., endoscope, ultrasound, cholangioscope) is selected on the video processor and our recording device directly captures this output, choosing a source other than the endoscope inadvertently includes unintended content in the recording, necessitating additional preprocessing. To ensure staff compliance and motivation, presence of a dedicated research assistant on-site to address questions or problems and regularly updating the team on project outcomes have proven valuable.

### Boston

Recording endoscopic procedures has become standard practice at BIDMC and the current implementation contributes over 100 videos to our database daily. The cloud-based Virgo system automates the recording process, eliminating the need for manual recording and minimizing delayed or missed recordings by automatically starting the recording upon insertion. However, because the AI for in-out recognition is not entirely reliable, some videos require manual cropping to ensure no identifiable visual information is present. Furthermore, the system does not capture other patient data. Nonetheless, videos are stored on proprietary servers, raising concerns regarding data protection and questions about data ownership. The process of manually matching electronic health records and recorded videos retrospectively remains time-consuming. Moreover, the inability to download multiple videos simultaneously from the Virgo Cloud and the lower-resolution storage diminish the benefits of advanced endoscope technology and increase the manual workload at BIDMC. Current limitations are being addressed in collaboration with industry and technical partners, and our workflows are continuously reviewed and improved.

### Würzburg

Our in-house developed system not only enables us to record the interventions from up to nine centers with diverse endoscopic processors, but it also facilitates anonymization and cropping of images, triggers the start and end of the recording, and detects images obtained by the endoscopist during the procedure. Moreover, it also allows us to expand system capabilities with new features, and rapidly validate novel AIs developed by our research group 9 10 . With this method, we have successfully collected more than 9000 endoscopic videos. The main limitation of our solution is the lack of integration with the EHR of the participating centers. Consequently, merging the collected data must be performed manually after the intervention. This introduces additional workload for our personnel but also ensures that stored videos are checked for errors prior to central storage.

### Privacy, data security, and standardization

All presented approaches successfully enable endoscopy video recording in clinical practice, each with its own advantages and limitations outlined, as described above. However, all are part of a broader discussion on privacy rights, data security, and ownership. In the case of locally implemented solutions like in Würzburg and Dresden, considerations regarding third parties handling the data (e.g., through cloud storage or similar) are not necessary. However, the question remains whether and under what conditions patient examinations can be recorded. Institutions planning to implement procedure video recording for scientific use should consult their local data security officer and seek approval from the ethics committee or institutional review board to jointly agree on a solution that serves the purpose of recording examinations and is in accordance with the relevant statutes. Although beyond the scope of this article, best practices for data security and privacy rights remain the focus of ongoing scientific debate 3 11 12 .

To maximize the usefulness of real-world data, some degree of standardization is necessary. Although efforts exist to harmonize EHR data (e.g., lab results), standardizing endoscopy-related data (especially examination reports) remains challenging. These reports are inherently unstructured and primarily designed for clinical care rather than research analysis, but methods like natural language processing could help to structure and harmonize these data at scale 13 . Nonetheless, video recordings alone, without accompanying metadata like annotations, demographics, or examination reports, already are a useful resource for unsupervised or self-supervised AI model development like foundation models 14 .

## Conclusions

In conclusion, routine recording of endoscopic examinations necessitates both financial and personnel investment during the initial implementation phase. However, once the workflow is established, the additional burden on on-site staff is minimal. Summarizing our experience in establishing video recording of endoscopic examinations in clinical practice, we recommend the following key steps for the planning and implementation phase: 1) Identify interprofessional collaborators early, seek their input, and address potential concerns; 2) Consult with the on-site medical technician to discuss setup characteristics in the local endoscopy unit and determine suitable recording strategies; 3) Consider medicolegal implications and obtain approval from the local ethics committee or institutional review board, ensuring patient privacy protection; 4) Assign a dedicated research member (e.g., a study nurse) to oversee daily operations and serve as a point of contact for any issues; and 5) Regularly update staff on project progress and results to maintain motivation. However, continuous evaluation and adoption of new solutions are crucial to enhance current processes. Although advanced recording setups can enhance workflow and recording quality, a basic setup consisting of a medical-grade recording device, a hard drive, and a digital spreadsheet is sufficient to quickly initiate endoscopic video recording. This practice empowers research groups to collect scientifically valuable data from routine clinical examinations, thereby contributing to the advancement of endoscopic research.
